# Research progress of radiation-induced hypothyroidism in head and neck cancer

**DOI:** 10.7150/jca.48587

**Published:** 2021-01-01

**Authors:** Ling Zhou, Jia Chen, Chang-Juan Tao, Ming Chen, Zhong-Hua Yu, Yuan-Yuan Chen

**Affiliations:** 1 Institute of Cancer and Basic Medical (ICBM), Chinese Academy of Sciences, Hangzhou, Zhejiang 310022, China.; 2 Department of Radiation Oncology, Cancer Hospital of University of Chinese Academy of Sciences, Hangzhou, Zhejiang 310022, China.; 3 Department of Radiation Oncology, Zhejiang Cancer Hospital, Hangzhou, Zhejiang 310022, China.; 4 The First Clinical Medical College, Guangdong Medical University, Zhanjiang, Guangdong 524000, China.; 5 Medical Research Institute, Hangzhou YITU Healthcare Technology Co., Ltd, Hangzhou, Zhejiang 330106, China.; 6 Shanghai Key Laboratory of Artificial Intelligence for Medical Image and Knowledge Graph, Shanghai 200050, China.; 7 Department of Oncology, the Affiliated Hospital of Guangdong Medical University, Zhanjiang, Guangdong 524001, China.

**Keywords:** Head and Neck Cancer, Hypothyroidism, Radiotherapy

## Abstract

This paper reviews the factors related to hypothyroidism after radiotherapy in patients with head and neck cancer to facilitate the prevention of radiation-induced hypothyroidism and reduce its incidence. Hypothyroidism is a common complication after radiotherapy in patients with head and neck cancer, wherein the higher the radiation dose to the thyroid and pituitary gland, the higher the incidence of hypothyroidism. With prolonged follow-up time, the incidence of hypothyroidism gradually increases. Intensity modulated radiotherapy should limit the dose to the thyroid, which would reduce the incidence of hypothyroidism. In addition, the risk factors for hypothyroidism include small thyroid volume size, female sex, and previous neck surgery. The incidence of radiation-induced hypothyroidism in head and neck cancer is related to the radiation dose, radiotherapy technique, thyroid volume, sex, and age. A prospective, large sample and long-term follow-up study should be carried out to establish a model of normal tissue complications that are likely to be related to radiation-induced hypothyroidism.

## Introduction

Head and neck cancer includes otolaryngological cancer, oral and maxillofacial cancer, and neck cancer, which account for approximately 6% of all malignant cancer worldwide 1. Radiotherapy is the main treatment modality for head and neck cancer, and the patients' neck is often included in the radiation field when receiving radiotherapy, resulting in damage to the normal tissue of the neck, especially radiation-induced hypothyroidism 2.

Hypothyroidism is divided into clinical and subclinical hypothyroidism. Clinical hypothyroidism is defined as having occurred when the concentration of thyroid stimulating hormone (TSH) is higher than the normal range, and the concentration of free thyroxine (FT4) is lower than the normal range. Subclinical hypothyroidism is defined as having occurred when the concentration of TSH is higher than the normal range, and the concentration of FT4 is in the normal range. According to its etiology, hypothyroidism can also be divided into primary, central, and peripheral hypothyroidism 3. One of the main causes of primary hypothyroidism is radiotherapy delivered to the head and neck that leads directly to thyroid dysfunction. Central hypothyroidism is associated with the hypothalamus-pituitary-thyroid axis, including hypothalamus or pituitary dysfunction, which is defined as a TSH concentration below the normal range and an FT4 concentration below normal range 4. Peripheral hypothyroidism refers to thyroid hormone receptor damage, resulting in thyroid hormone dysfunction. Central and peripheral hypothyroidism is rare, with an incidence of less than 1% 5. The common clinical symptoms of hypothyroidism are fatigue, drowsiness, intolerance of cold, weight gain, constipation, aural changes, and dry skin 6. Mild cases have no obvious symptoms, while severe cases can have an increased incidence of cardiovascular diseases, such as heart failure, atrial fibrillation, coronary heart disease, and the risk of death 7-9, which varies according to age, sex, and duration of symptoms.

The mechanism of radiation-induced hypothyroidism has not been fully clarified; however, it is related to thyroid cell fibrosis, vascular proliferation, and the immune response 10-12. Studies have shown that radiation can damage thyroid cells directly, interfere with their mitosis, affect the blood supply, or induce a certain immune response 13. Lin et al. 14 believed that hypothyroidism is closely related to thyroid antibodies. The increased expression of anti-thyroid peroxidase and anti-thyroglobulin antibodies in patients after radiotherapy suggested that an immune response might be one of the mechanisms of radiation-induced hypothyroidism.

In recent years, with the application of intensity modulated radiotherapy (IMRT), the survival time of patients has been prolonged significantly; therefore, their quality of life has received widespread attention 15-16. Patients with head and neck lymphoma 17-18, central nervous system tumors 19-20, and head and neck cancer 21-22 require routine radiotherapy, and the pituitary or thyroid is included in the radiation field, resulting in a high incidence of hypothyroidism. Since Felix first discovered radiation-induced hypothyroidism in patients with laryngeal carcinoma in 1961 23, there have been many studies on the factors related to radiation-induced hypothyroidism in head and neck cancer. Clinical factors such as dose-volume parameters 24-26, chemotherapy 2,27-28, neck surgery 29-30, sex 2,27,31, age 2,24,32, and follow-up time 33, are considered risk factors for radiation-induced hypothyroidism. At the same time, models of normal tissue complication probability (NTCP) related to radiation-induced hypothyroidism in head and neck cancer were established according to the risk factors; however, the related factors included in the models remain controversial. This article reviews the factors related to radiation-induced hypothyroidism in head and neck cancer, including radiotherapy, chemotherapy, surgical treatment, other clinical factors, and NTCP models.

## Radiotherapy

### Thyroid dose-volume thresholds

It is reported that the predicted 5-year risk of dysfunction is 5% with a 45-Gy dose and 50% with an 80-Gy dose 34. With the development of radiotherapy technology, the dose threshold related to radiation-induced hypothyroidism in patients with head and neck cancer needs to be further explored. The International Journal of Radiation Oncology Biology Physics published guidelines for the quantitative analysis of normal tissue effects in the clinic (QUANTEC) in 2010, including safe doses for the brainstem, spinal cord, optic nerve, and other tissues; however, they did not mention the thyroid 35. In recent years, differences in tumor location, the definition of radiation-induced hypothyroidism, treatment methods, and follow-up time, in studies that explored the optimal thyroid dose-volume threshold have meant that no consensus has been reached.

Most studies included several non-thyroid cancers in their definition of head and neck cancers, such as nasopharyngeal, oral, laryngeal, and hypopharyngeal cancers. The incidence of radiation-induced hypothyroidism in patients with head and neck cancer is 20-60%, which increases with time, as shown in **Table 1.** Boomsma et al. 36 considered that the incidence of hypothyroidism increased with the decrease in thyroid volume and the increase in the mean radiation dose; however, the optimal threshold dose for the thyroid was not determined. Fujiwara et al. 37 found that the mean dose, VS10 (VSx: The volume of the thyroid gland spared from doses ≥ x Gy), VS20, VS30, and VS40 of thyroid were closely related to hypothyroidism. Among them, the mean dose (Dmean) was an independent predictor, and Dmean ≤ 30 Gy could protect thyroid function. Kim et al. 38 analyzed 114 patients with head and neck cancer retrospectively and showed that the thyroid dosimetric parameter V45 (Vx: the percentage of thyroid volume receiving > x Gy) was an independent predictor of hypothyroidism. The 1-year cumulative incidences of hypothyroidism in the group with V45 < 50% and V45 ≥ 50% were 22.8% and 56.1% respectively, and the difference was statistically significant. Murthy et al. 39 analyzed 44 patients with head and neck cancer who received 3D conformal radiotherapy (3D-CRT) and 45 patients who received IMRT. They found that a thyroid dose less than 40 Gy could reduce the incidence of hypothyroidism. All of the above studies included patients who received 3D-CRT; therefore, the results are not fully applicable to patients receiving IMRT.

Prpic et al. 40 analyzed retrospectively 156 patients with head and neck cancer treated with IMRT and showed that thyroid dose parameters VS65, minimum dose (Dmin), and V50 were related to the incidence of hypothyroidism. Sachdev et al. 41 suggested that thyroid V50 < 60% should be set as the optimal dose parameter. Zhai et al. 42 analyzed 135 patients prospectively, and univariate analysis showed that Dmin, Dmean, V30, V35, V40, V45, and V50 of the thyroid were associated with hypothyroidism, among which Dmean, V45, and V50 were independent predictors. The relative risk of hypothyroidism for patients with Dmean > 45 Gy was 4.9 times higher than that for patients receiving a lower dose, and the authors recommended that the thyroid dose be set to V45 < 50% and V50 < 35%. Similarly, Xu et al. 43 found that the thyroid dose parameters Dmean and V50 in the hypothyroidism group were significantly higher than those in the normal group. The 3-year cumulative incidences of radiation-induced hypothyroidism in the group with Dmean < 5160 cGy and Dmean > 5160 cGy were 44.6% and 67.8% respectively (*P* = 0.036). The lower incidence group had a V50 < 54.5% and the higher incidence group had a V50 > 54.5% (29.9% *vs*. 66.1%, *P* = 0.025). Sommat et al. 24 found that the incidences of hypothyroidism in patients with V40 ≤ 85% and V40 > 85% were 21.4% and 61.4% respectively. The authors recommended that the thyroid dose-volume threshold should be set to V40 ≤ 85%. Compared with other studies, this dose limit is not strict. Huang et al. 44 believed that thyroid dose parameters should be individually limited, such as V25 ≤ 60%, V35 ≤ 55%, and V45 ≤ 45%, which could strictly control the thyroid dose and reduce the incidence of hypothyroidism, making it suitable for patients with a small tumor and those at the early clinical stages. They noted that V25 > 95%, V35 > 90%, and V45 > 75% are risk factors for hypothyroidism and should be avoided as far as possible. VSx parameters may be more suitable than Vx parameters to predict the incidence of radiation-induced hypothyroidism, because the thyroid is a parallel organ, and the number of thyroid follicular cells not exposed to high dose radiation is an important factor to produce thyroid hormones and maintain normal metabolism. Under the conditions that the radiation field includes tumors completely, VS60 ≥ 10 cm^3^ and VS45 ≥ 5 cm^3^ could be regarded as the primary and secondary principles of thyroid dose limitation 25. Lertbutsayanukul et al. 26 also believed that VS60 ≥ 10 cm^3^ is a protective factor of thyroid function. The wider use of thyroid dose-volume parameters has been limited by the differences in various research conditions. A meta-analysis that included five articles related to radiation-induced hypothyroidism 21-22,32,45-47 found that thyroid dose-volume parameters V10-V70, Dmean, Dmin, and maximum dose (Dmax) to the thyroid were related to hypothyroidism; however, the optimal dose threshold of the thyroid could not be determined.

Most studies proposed that the thyroid volume is related to hypothyroidism. The smaller the thyroid volume, the higher the incidence of hypothyroidism 25,36,42. Lin et al. 48 observed that the thyroid volume in patients with nasopharyngeal carcinoma showed a decreasing trend after radiotherapy; the trend was more obvious after 6 months, and then gradually slowed down. Boomsma et al. 36 considered that the relationship between the incidence of hypothyroidism and thyroid volume was 5%/cm^3^, for every 1 cm^3^ decrease in thyroid volume, the incidence of hypothyroidism increased by 5%. However, Diaz et al. 32 believed that for every 1 cm^3^ decrease in thyroid volume, the incidence of hypothyroidism increases by 7%. Chyan et al. 49 analyzed 123 patients with oropharyngeal carcinoma retrospectively and found that the thyroid volume threshold associated with radiation-induced hypothyroidism was 8 cm^3^. This result might have been affected by confounding factors in the study and is not an exact biological threshold; however, it suggests that thyroid volume affects dose limitation. For patients whose thyroid volume is larger than 8 cm^3^, setting the thyroid dose to D3CC < 45Gy (at least 3 cm^3^ of the thyroid can be spared from doses exceeding 45 Gy) could reduce the incidence of hypothyroidism. If the volume of thyroid is less than 8 cm^3^, more strict restrictions are required, such as Dmean < 49 Gy, V50 < 45%, VS45 ≥ 3 cm^3^, and VS50 ≥ 6 cm^3^.

### Pituitary dose-volume thresholds

The radiation field of patients with head and neck cancer receiving radiotherapy often includes the hypothalamus and pituitary, which can cause central hypothyroidism through the hypothalamus-pituitary-thyroid axis, especially in patients with nasopharyngeal carcinoma. Huang et al. 50 found that the expression level of thyroid hormone in patients with nasopharyngeal carcinoma was most closely related to the dose-volume parameter V55 of the pituitary. Lin et al. 51 explored the effect of thyroid and pituitary radiation dose on thyroid function. The patients were divided into four groups: High (>50 Gy) thyroid and pituitary doses (HTHP group), high thyroid and low pituitary doses (HTLP group), low thyroid and high pituitary doses, and low thyroid and pituitary doses. The results showed that the HTHP group had the highest incidence (83.3%) of hypothyroidism, followed by the HTLP group (50%), which confirmed that radiation would damage the pituitary and thyroid function, but had little effect on the pituitary gland. Central hypothyroidism caused by pituitary dysfunction is rare and mild, which is easily ignored by clinicians. To date, most studies have not found central hypothyroidism 24-25,42, which might be the result of insufficient follow-up time. The incidence of central hypothyroidism in head and neck cancer is 5.4%, and the clinical median latency period is 4.8 years 22.

Luo et al. 52 analyzed 164 patients with nasopharyngeal carcinoma prospectively and found that the Dmean and Dmax of the pituitary were 30.366 Gy and 37.856 Gy, respectively. The results showed that the pituitary Dmax was associated with hypothyroidism. Xu et al. 43 found that the dose-volume parameters Dmean, V30, V40, V50 and V55 of the pituitary were not significantly correlated with hypothyroidism; however, the pituitary dose was higher in the hypothyroidism group. Ratnasingam et al. 53 assessed the endocrine function of patients after radiotherapy, and found that 82% of the patients developed pituitary dysfunction, and the incidence of adrenocorticotrophic hormone, gonadotropic hormone, and thyroid stimulating hormone deficiency were 40%, 22%, and 4%, respectively. Even if the incidence of central hypothyroidism is low, the effect of pituitary radiation dose on endocrine function in patients receiving radiotherapy cannot be ignored.

### Radiotherapy technique

With the progress of radiotherapy technology, the incidence of side effects in patients with head and neck cancer receiving radiotherapy has decreased. IMRT can better protect normal organs than 3D-CRT 54-55. Luo et al. 52 analyzed retrospectively 32 patients who received 3D-CRT and 142 patients who received IMRT retrospectively and observed an incidence of biochemical hypothyroidism in patients of 22.4%. Fujiwara et al. 37 studied 116 patients with head and neck cancer who received 3D-CRT and noted an incidence of hypothyroidism of 38.6% (39/116). Among them, 21 cases had clinical hypothyroidism, which was a much higher incidence than that reported by other studies 25. This suggested that 3D-CRT results in higher levels of side effects in normal tissues; however, there were differences in methods, endpoints, and follow-up times among the studies, which could have introduced bias into the comparison of the incidence of side effects.

Diaz et al. 32 compared the effects of radiotherapy techniques on the incidence of hypothyroidism and found that the thyroid dose parameters V10, V20, V30, Dmin, Dmax, and Dmean in patients receiving 3D-CRT were significantly lower than those in patients receiving IMRT without limiting the thyroid dose. After limiting the thyroid dose, using IMRT could significantly reduce the median dose. Murthy et al. 39 compared 44 patients with head and neck cancer who received 3D-CRT and 45 patients who received IMRT, and found that the incidence of hypothyroidism in the IMRT group was higher than that in the 3D-CRT group (64.4% *vs*. 45.5%), possibly because the middle of the neck was not irradiated in the 3D-CRT group, while in the IMRT group, the thyroid dose was not limited. In 3D-CRT group, the thyroid radiation dose delivered to the patients who developed hypothyroidism was higher than that in the euthyroid group; however, there was no correlation between thyroid dose-volume parameters and the incidence of hypothyroidism in the IMRT group. In summary, the thyroid dose must be limited during IMRT to reduce the thyroid radiation dose as far as possible, which will reduce the incidence of hypothyroidism.

## Chemotherapy

It is controversial whether chemotherapy increases the incidence of radiation-induced hypothyroidism. Most studies proposed that there is no correlation between chemotherapy and hypothyroidism 25,52,56. A meta-analysis showed that female sex, neck surgery, being Caucasian, and receiving lymphangiography are risk factors for hypothyroidism, while chemotherapy did not affect the incidence of hypothyroidism 57. Fan et al. 2 showed that chemotherapy is an independent risk factor for radiation-induce hypothyroidism, which was consistent with the results of Ling et al. 31. Hypothalamus-pituitary dysfunction often occurs in patients after radiotherapy, resulting in central hypothyroidism, for which concurrent chemotherapy is a risk factor, which increases with time. To date, there have been few studies investigating whether chemotherapy is related to radiation-induced hypothyroidism, and there is no clear conclusion on the role of chemotherapy in hypothyroidism.

## Surgical treatment

Alba et al. 58 found that laryngeal surgery is a risk factor for hypothyroidism through an analysis of 241 patients with head and neck cancer. Surgery produces pressure on the thyroid, and treatment and ligation of blood vessels affect the blood supply of the thyroid, leading to thyroid dysfunction during radiotherapy 59. A cohort study by Lin et al. 60 found that primary tumor resection combined with neck surgery was associated with a higher risk of thyroid disease in patients with hypopharyngeal, oropharyngeal, and laryngeal cancers. Neck surgery increased the risk of radiation-induced hypothyroidism, which might be caused by skin fibrosis and arteriosclerosis after surgery. A meta-analysis showed that whether the scope of surgery involves thyroid or not, it will increase the risk of hypothyroidism 57. However, other studies suggested that there is no significant correlation between neck surgery and hypothyroidism 13,38; therefore, the effect of neck surgery on hypothyroidism should be further explored. The thyroid function of patients with head and neck cancer who have undergone surgery should be closely observed after radiotherapy, especially in patients with laryngeal cancer, hypopharyngeal cancer, and other tumors close to the thyroid gland.

## Other clinical factors

### Sex

Ronjom et al. 61 found that the median thyroid volume of women was significantly lower than that of men (13.4 cm^3^ *vs*. 18.4 cm^3^) (*P* < 0.001); however, there was no significant relationship between thyroid volume and sex, even if both of them were significant predictors of hypothyroidism (*P* < 0.008). Ling et al. 31 considered that a woman's thyroid gland was smaller (12.5 cm^3^ *vs*. 15 cm^3^) and more prone to developing hypothyroidism than a man's thyroid; however, there was no significant difference in the thyroid Dmean (50.9 Gy *vs*. 48.8 Gy) between women and men. Fan et al. 2 found that the incidence of hypothyroidism in female patients was 2.03 times higher than that in male patients via analysis of a large sample. Lertbutsayanukul et al. 26 analyzed 178 patients prospectively, in which univariate analysis showed that a small thyroid volume (< 8 cm^3^) was a risk factor for hypothyroidism, and multivariate analysis showed that thyroid VS60 ≥ 10 cm^3^ and pretreatment TSH < 1.55 μU/ml could protect thyroid function. This was similar to the results of Zhai et al. 42, who believed that younger age, female sex, and a smaller thyroid volume are significantly associated with hypothyroidism. Although the relationship between sex and thyroid volume remains to be further determined, most studies believed that women are more likely to develop hypothyroidism 57. Therefore, the endocrine function in female patients should be followed up closely after radiotherapy.

### Age

Many studies have shown that the incidence of hypothyroidism is related to age, with children and adolescents being more sensitive to radiation and more prone to developing hypothyroidism 24,36,62. By contrast, some scholars have found no correlation between age and hypothyroidism 13,25,56; however, the difference in age boundary values was one of the main reasons for disagreement. Huang et al. 44 showed that younger age (≤ 44 years old) was associated with hypothyroidism, while age was not significantly associated in multivariate analysis. One study found that the independent risk factors for radiation-induced hypothyroidism included younger age; the incidence of hypothyroidism decreased 0.9 times with every increase in age 2. Similarly, Diaz et al. 32 found that the incidence of hypothyroidism decreased by 4% with every increase in age. In addition, Smith et al. 63 analyzed 5916 elderly patients (> 65 years old) with head and neck cancer and found that the risk of hypothyroidism in elderly patients after radiotherapy was twice as high as that in patients who only received surgery, with the risk of hypothyroidism lasting until 10 years after radiotherapy. Tell et al. 30 reported that older age increased the risk of subclinical hypothyroidism, but did not affect the incidence of clinical hypothyroidism, which might have been the result of the older median age of the patients in this study (65 years old). Thyroid function should be continuously monitored after radiotherapy for children or elderly patients, and elderly patients should be followed up for their lifetime.

### Clinical stage

The radiation field of head and neck cancer is related to the tumor-node-metastasis (TNM) stage. Studies have reported that early T stage is a risk factor for radiation-induced hypothyroidism 24 62, which seemed to be controversial. Wu et al. 62 reported that radiotherapy-induced damage to the pituitary gland prohibited the response of the pituitary gland to low levels of serum thyroid hormones in patients with advanced stage (T3-4) nasopharyngeal carcinoma. Theoretically, the radiation field in patients with T3-4 nasopharyngeal carcinoma includes the skull base, resulting in a high pituitary dose and a tendency to develop central hypothyroidism. This discrepancy might have been caused by the research endpoints not including central hypothyroidism. Other studies have found no correlation between T stage and the incidence of hypothyroidism 43.

Fujiwara et al. 37 analyzed 116 patients with head and neck cancer who received 3D-CRT, and univariate analysis showed that the incidence of hypothyroidism was significantly higher in the positive cervical lymph node group. Positive lymph nodes increase the exposure volume of primary tumors and the risk of thyroid irradiation; therefore, positive lymph node status is an important factor for hypothyroidism 39. However, Koc et al. 13 believed that there is no correlation between clinical stage and hypothyroidism, which might have been affected by some confounding factors. Attention should be paid to patients with advanced N stage during individualized follow up of thyroid function test.

### Pretreatment TSH values

The diversity in the diagnosis of hypothyroidism means that there are differences in the normal range of TSH. To date, few studies have considered the baseline level of TSH. Lertbutsayanukul et al. 26 reported that a high pretreatment TSH value (≥ 1.55 μU/ml) was a risk factor for radiation-induced hypothyroidism. Ronjom et al. 61 included 203 patients with head and neck cancer non-nasopharyngeal carcinoma, and showed that although the thyroid volume was a significant predictor of hypothyroidism, the pretreatment TSH value was not related to hypothyroidism. However, there was a significant correlation between the pretreatment TSH value and thyroid volume: Patients with a smaller thyroid volume had higher pretreatment TSH values. In that study, in addition to the traditional definition of hypothyroidism (TSH > 4.0 mIU/L), the pretreatment TSH value was used to define hypothyroidism for the first time, comprising an increase in TSH from baseline of ΔTSH > 2.7 mIU/l. This threshold change was chosen such that the number of patients with hypothyroidism was identical to the number achieved based on an absolute TSH level above 4 mIU/l. The results obtained using the external validation group showed that the pretreatment TSH value was closely related to hypothyroidism 64, which was slightly different from previous results, because most patients in the validation group had their blood collected within two weeks before treatment; however, the decrease in TSH was an acute reaction to cervical radiotherapy 65.

### NTCP models

To date, a large number of studies have aimed to restrict the individualized thyroid dose and establish a NTCP model to predict the incidence of radiation-induced hypothyroidism. Boomsma et al 36 studied an NTCP model of radiation-induced hypothyroidism prospectively for the first time. They found that the independent predictors of hypothyroidism were thyroid Dmean and volume. The incidence of hypothyroidism increased with increasing Dmean and decreased with increasing volume. The NTCP model with Dmean and volume has shown the best performance (area under the curve (AUC) = 0.85). However, Ronjom et al. 61 reported that there was no obvious interaction between Dmean and volume. Consistent with the above research, the NTCP model established using Dmean and volume had a better effect, and an external group confirmed that a higher Dmean and smaller thyroid volume were important risk factors for radiation-induced hypothyroidism in head and neck cancer 64. In addition, they observed that the differences in thyroid contours also affect the performance of the NTCP model. There are large differences in assessing the risk of hypothyroidism for different contours, which requires clinicians to accurately sketch the outline of the thyroid to obtain a correct risk assessment, permitting individualization of the treatment plan 66. Bakhshandeh et al. 67 assessed six established NTCP models, including phenomenological and structural models, to explore the thyroid dose-volume relationship related to radiation-induced hypothyroidism in head and neck cancer. Ultimately, they confirmed that the NTCP model is closely related to the Dmean of the thyroid. The model including Dmean is the simplest, and this NTCP model has a strong correlation with the thyroid volume. Luo et al. 52 showed that sex, chemotherapy, thyroid V50, and pituitary Dmax were related to hypothyroidism in a prospective study of 174 patients with nasopharyngeal carcinoma. The predictive effect in patients with nasopharyngeal carcinoma was better than that in patients with other head and neck cancers when the above four factors were included in the NTCP model (AUC = 0.793), probably because the model included pituitary dosimetry factors. The effects of radiotherapy on the pituitary gland and thyroid gland should be considered when establishing radiation-induced hypothyroidism NTCP models in patients with nasopharyngeal carcinoma.

## Conclusion

Research has shown that radiation-induced hypothyroidism is related to radiotherapy, (including thyroid dose-volume thresholds, pituitary dose-volume thresholds, and radiotherapy technique), chemotherapy, surgical treatment, and other clinical factors (such as sex, age, clinical stage, and pretreatment TSH value). Thyroid function should be closely monitored in patients at high risk of hypothyroidism, such as those receiving a higher thyroid dose and a pituitary dose; those with a smaller thyroid volume; those receiving chemotherapy or surgery; and those who are younger, female, have advanced stage disease, and a higher pretreatment TSH value. Even if there is no unified standard for the optimal thyroid threshold, it is still necessary to limit the thyroid dose while ensuring coverage of primary tumor. In the future, a prospective, large sample and long-term follow-up study should be carried out to establish a better NTCP model related to radiation-induced hypothyroidism and to individually limit the thyroid dose. At the same time, further study of the mechanism of radiation-induced hypothyroidism, exploration of the relationship between radiotherapy and hypothyroidism, achievement of early prevention and early treatment, a reduction in the incidence of radiation-induced hypothyroidism, and improvement of the quality of life of patients with head and neck cancer should be undertaken.

## Figures and Tables

**Table 1:** Incidence of radiation-induced hypothyroidism in head and neck cancer

Authors	Tumor type	6 m	12 m	18 m	24 m	30 m	36 m	Median time
Sommat et al. 24	NPC	-	33%	-	44.5%	-	-	36.7 m
Lee et al. 25	NPC	-	5.3%	-	17.5%	-	36.2%	-
Lertbutsayanukul et al. 26	NPC	-	21.6%	-	43.6%	-	56.6%	21 m
Luo et al. 27	NPC	6.8%	14.3%	22.2%	24.6%	26.6%	-	10.5 m
Fujiwara et al. 37	HNC	-	21.1%	-	36.4%	-	48.3%	21 m
Zhai et al. 42	NPC	-	13.2%	-	29.6%	-	43.9%	15.1 m
Huang et al. 44	NPC	-	10.7%	-	25.8%	-	25.8%	-

Abbreviations: m: month; NPC: nasopharyngeal carcinoma; HNC: head and neck cancer; Median time: the median time to develop hypothyroidism.

**Table 2:** Factors related to radiation-induced hypothyroidism in head and neck cancer

Authors	N	Tumor type	Median follow-up time	Radiotherapy type	Incidence of biochemical HT	Incidence of Clinical HT	Related clinical factors	Dosimetric factor
Sommat et al. 24	102	NPC	48.8 m	IMRT	55.9%	16.7%	Age, T stage	V40 ≤ 85%
Lee et al. 25	149	NPC	3.1 y	IMRT	22.1%	14.1%	-	V, VS60, VS45
Lertbutsayanukul et al. 26	178	NPC	42.5 m	IMRT	53.9%	-	Pretreatment TSH	VS60 < 10 cm3
Luo et al. 27	164	NPC	24 m	3D-CRT, IMRT	22.4%	-	Gender, Chemotherapy	Thyroid V50, Pituitary Dmax
Ling et al. 31	102	HNC	33.5 m	3D-CRT, IMRT	39.2%	30.4%	Gender, Chemotherapy	D50% < 50Gy, V50 < 50%, Dmean < 54.58 Gy
Boomsma et al. 36	105	HNC	2.49 y	3D-CRT, IMRT	33.3%	8.6%	Thyroid volume	Dmean
Fujiwara et al. 37	116	HNC	24 m	3D-CRT	38.6%	20.8%	*ENI	Dmean ≤ 30Gy, VS10, VS20, VS30, VS40
Kim et al. 38	114	HNC	25 m	3D-CRT, IMRT	36.0%	9.6%	-	V45 <50%
Murthy et al. 39	89	HNC	41 m	3D-CRT, IMRT	55.1%	15.7%	Age, Tumor location, Node positivity, Dose/fraction (IMRT arm)	D100<40 Gy
Prpic et al. 40	156	HNC	23 m	-	44.9%	-	-	Thyroid volume, Dmin
Sachdev et al. 41	75	HNC	50 m	IMRT	-	33%	-	V50 ≤ 60%
Zhai et al. 42	135	NPC	34.1 m	IMRT	28.9%	-	Age, Thyroid volume	Dmean <45 Gy, V45 < 0.50, V50 < 0.35
Huang et al. 44	345	NPC	45.2 m	IMRT	44.1%	11.0%	Pretreatment thyroid volume	V25 ≤ 60%, V35 ≤ 55%, V45 ≤ 45%

Abbreviations: N: the number of patients; NPC: nasopharyngeal carcinoma; HNC: head and neck cancer; m: month; y: year; 3D-CRT: 3-dimensional conformal radiotherapy; IMRT: intensity-modulated radiation therapy; *ENI: Elective node irradiation; Dmean: Mean dose to thyroid; Dmin: Minimum dose to thyroid; D100: Dose to 100% of thyroid; VSx: The volume of the thyroid gland spared from doses ≥ x Gy. Vx(%): the percentage of thyroid volume receiving > x Gy; TSH: thyroid stimulating hormone.
